# Pathogen-pathogen interactions during co-infections

**DOI:** 10.1093/ismejo/wraf104

**Published:** 2025-05-23

**Authors:** Rosana Barreto Rocha Ferreira, Luis Caetano Martha Antunes, Neta Sal-Man

**Affiliations:** Department of Molecular Biosciences, University of Kansas, 1200 Sunnyside Avenue, Lawrence, KS 66045, United States; Department of Molecular Biosciences, University of Kansas, 1200 Sunnyside Avenue, Lawrence, KS 66045, United States; The Shraga Segal Department of Microbiology and Immunology, Ben-Gurion University of the Negev, Beer Sheva, Israel

**Keywords:** bacterial competition, bacterial pathogens, competition avoidance, pathogen cooperation, polymicrobial infection, polymicrobial infection therapy

## Abstract

For over a century, bacterial infections have been studied through the lens of the one-microbe, one-disease paradigm. However, it is now clear that multi-pathogen infections are common, and many infectious diseases are inherently polymicrobial. These complex infections can involve a variety of pathogens, including viruses, bacteria, fungi, and parasites, with polyviral and viral-bacterial interactions being the most extensively studied. In this review, we focus on polybacterial infections, providing an in-depth analysis of the diverse strategies bacteria employ to thrive in co-infection scenarios. We examine the mechanisms of bacterial competition, competition avoidance through spatial or temporal separation, and cooperation. Given the association of polymicrobial infections with more severe clinical outcomes and heightened antibiotic tolerance, we also explore novel therapeutic targets to treat these increasingly common and complex infections. Although our review summarizes current knowledge, the vast scope of this phenomenon suggests that many more mechanisms remain undiscovered and warrant further investigation.

## Introduction

Following Koch's postulates, linking single microbes to specific diseases, infectious disease research has focused on single pathogens growing in isolation (see [1] for a modern take on Koch’s postulates). This approach has proven instrumental in advancing knowledge by determining disease etiology, thereby promoting diagnosis, prevention, and treatment strategies. However, likely due to improved diagnostic techniques, the percentage of infections found to be polymicrobial (involving two or more infectious agents) has been on the rise, reaching up to 40% of pediatric clinical samples [2–7]. Nevertheless, the true prevalence of polymicrobial infections is likely underestimated, as diagnostic protocols are designed mainly for mono-infections and the likelihood of detecting polymicrobial infections varies based on the techniques employed and the clinician’s intent [8]. Although certain diseases (periodontitis, otitis media, urinary tract infections (UTIs)), are well-established as polymicrobial [9, 10], others are traditionally considered monomicrobial, with diagnosis focusing on the dominant pathogen. However, there is a growing recognition that many infectious diseases are multifactorial, involving multiple pathogens.

Theoretical models comparing monomicrobial and polymicrobial infections predict the latter result in greater host damage [11, 12]. Indeed, previous work showed polymicrobial infections can be associated with more severe clinical symptoms, possibly due to increased overall infectious burden or heightened virulence of at least one of the pathogens, and are often more tolerant to antibiotics [4, 13–18]. However, the outcome of polymicrobial infections is shaped by both the infectious agents and host context, making it difficult to predict how these interactions will unfold. Under some conditions, polymicrobial infections may be just as, or even less, severe than mono-infections. Regardless, the observation that, under some circumstances, polymicrobial infections can result in increased virulence emphasizes the need for clear criteria in diagnosing and treating such polymicrobial infections, likely leading to better patient outcomes [19, 20].

Although polymicrobial infections can involve viruses, bacteria, fungi, or parasites, this review focuses specifically on polybacterial infections. We discuss how multiple bacterial pathogens detect one another and modulate their behavior to either collaborate or compete. Also, it was not our goal to dissect the interactions that occur between pathogens and commensal microbes. Due to the complexity of the human microbiome, pathogens are constantly interacting with commensals, with significant implications for infection dynamics. However, this is beyond the scope of this review, and our focus is on the less explored realm of pathogen-pathogen interactions.

## Polybacterial infections in humans

Polybacterial infections occur at various body sites, including the respiratory tract, gastrointestinal system, and skin [21–23]. Often, specific bacterial pairs are found together, suggesting these associations are not random. In skin and soft tissue infections (SSTIs), such as chronic wounds, pressure ulcers, and burns, polymicrobial infections are particularly prevalent [21]. Bacterial species associated with polymicrobial SSTIs include *Staphylococcus*, *Enterobacteriaceae*, *Pseudomonas aeruginosa*, *Enterococcus*, *Acinetobacter baumannii*, *Streptococcus*, and strict anaerobes, with *Staphylococcus aureus* being the most frequent agent [21]. Due to the high incidence of methicillin-resistant *S. aureus* (MRSA), these infections tend to show more severe clinical manifestations and limited treatment options [24, 25]. Most *S. aureus*-associated SSTIs are polymicrobial, with *P. aeruginosa*, *Enterococcus faecalis*, *A. baumannii*, *Corynebacterium* spp., or *Escherichia coli* being co-isolated [26–29]. In diabetic foot ulcers, *S. aureus* is most frequently isolated alongside Gram-negative bacteria (*P. aeruginosa*, *Acinetobacter*, *E. coli*, *Enterobacter*, *Citrobacter*, *Proteus*, and *Klebsiella*), although some Gram-positive bacteria, such as *Enterococcus*, are also co-isolated [26, 30]. In pressure ulcers, *S. aureus* is frequently co-isolated *with P. aeruginosa*, *E. coli*, *P. mirabilis*, *Enterobacter cloacae*, and *E. faecalis* [31, 32], and, in burn wounds, with *P. aeruginosa* [33].

In the oral cavity, another known site for polymicrobial infections, *Porphyromonas gingivalis*, *Treponema denticola*, *Tannerella forsythia*, and *Fusobacterium nucleatum* are usually co-isolated [22, 34, 35]. These infections typically lead to periodontal disease, potentially causing tooth loss. Although bacteria directly harm tissues, damage is mostly by triggering host cell activation, leading to production of tissue-degrading substances. Although most research on periodontal disease uses single-species infection models, recent studies adopted polybacterial models, better mimicking natural disease [22, 34, 35]. In a murine co-infection abscess model with *F. nucleatum* and *P. gingivalis*, pathogen ratios and timing of infection were critical determinants of disease severity [34]. Higher proportions of *F. nucleatum* significantly reduced lesion formation. Other infection protocols, such as simultaneous inoculation with both pathogens at different sites, increased lesion formation by a trypsin-like protease of *P. gingivalis* [34]. These data suggest intricate interactions between pathogens, also likely involving the host immune system, are at play.

UTIs have historically been associated with single pathogens, like uropathogenic *E. coli* [36]. However, polybacterial infections are frequent in specific populations, such as individuals using urinary catheters, the elderly, and the immunocompromised [37]. Assessment of polymicrobial UTIs is complicated by sample contamination with periurethral and vaginal microbes [36]. Nevertheless, up to 86% of catheter-associated UTIs are polymicrobial [38]. The pathogens involved include *E. coli, Klebsiella pneumoniae, P. aeruginosa, Proteus mirabilis,* and *Providencia stuartii.* Among these, *P. mirabilis* and *P. stuartii* are consistently co-isolated from catheter-associated UTIs and increase urolithiasis and bacteremia in mice [38]. A recent analysis of urine and catheter colonization in long-term catheterized individuals revealed that long-term co-isolation was observed with uropathogenic *E. coli* and *E. faecalis* [39]. *In vitro* data, using artificial urine media, showed co-culturing with *E.coli* increases *E. faecalis* growth.

Although viruses are the primary cause of respiratory diseases, several bacteria can lead to severe illness, including *Streptococcus pneumoniae*, *Streptococcus pyogenes*, *Haemophilus influenzae*, *S. aureus*, *Neisseria meningitidis*, *Mycobacterium tuberculosis*, and *Bordetella pertussis*. Polymicrobial respiratory infections are widely reported, but consist mainly of viral-bacterial infections, where bacterial agents are considered secondary to initial damage caused by viruses [40]. Polybacterial lung infections are most commonly observed in patients with severe conditions, such as patients who underwent major surgery, chemotherapy and radiotherapy patients, and patients with the genetic disease cystic fibrosis (CF). In CF, infections often include *S. aureus*, *H. influenzae*, *P. aeruginosa*, and *Burkholderia cepacia* [41]. The interplay between these species in CF is intricate and dynamic. Some species are associated with poor clinical outcomes, whereas others are not. Ratios between different species vary widely and display longitudinal variation, also being affected by host factors, such as age [42].

Detecting polybacterial infections in the gastrointestinal tract is challenging due to the dense microbiome that populates this environment, making it difficult to evaluate the impact of commensals during infection. Also, several gut commensals can act as pathogens depending on the context (barrier breach, microbial density, acquisition of virulence genes through horizontal gene transfer). Interactions between gut commensals and classic enteric pathogens are common, and play significant roles in microbial virulence and disease severity. A healthy intestinal microbiome is associated with host colonization resistance to pathogens, adding complexity to pathogen-pathogen interactions [43]. A study of two cohorts of infants in Bangladesh found that two pathogen pairs, Enterotoxigenic *E. coli* (ETEC) with Enteropathogenic *E. coli* (EPEC) and ETEC with *Campylobacter* spp., appeared together more frequently than expected at random, suggesting these pairs are associated with gastrointestinal coinfections [44]. Additional studies showed multiple enteric pathogens can be detected in diarrheal samples, many of which carry ETEC and *Vibrio cholerae* [4, 6, 45]. Patients with these co-infections tend to experience more days with diarrhea, worse dehydration, and a need for higher intravenous fluid intake.

Interactions between bacterial species in complex communities can span from cooperation to competition, and this is likely also the case for pathogen-pathogen interactions [46]. In some cases, bacteria compete for resources and niche dominance, inhibiting each other's growth and colonization. Other scenarios involve cooperation, where different species support each other's survival and virulence through metabolic cross-feeding and biofilm formation [46]. These interactions impact the progression and severity of infections, underscoring the need to understand their underlying dynamics to develop more effective treatment strategies.

### Challenges in the diagnosis of polymicrobial infections

Currently, several methods are available for diagnosing monomicrobial infections, with culture-based techniques regarded as the gold standard [47]. Culture-based methods are cost-effective, require minimal specialized equipment and training, and uniquely allow for the detection of viable bacteria, which is essential for phenotypic antimicrobial susceptibility testing. However, they are time-consuming, require specific growth conditions for fastidious strains, and are often ineffective once antibiotic treatment has begun. In polymicrobial infections, traditional culture-based techniques are inherently limited, often selectively detecting the most abundant or the fastest-growing species. This methodological bias can lead to incomplete identification of the causative agents of these infections [48]. Molecular methods involving DNA sequencing, DNA/protein fingerprinting technology, and target-specific testing, such as multiplex PCR, digital PCR, Peptide Nucleic Acid Fluorescence *In Situ* Hybridization (PNA-FISH), and Molecular Antimicrobial Susceptibility Testing (AST) have significantly enhanced the diagnosis of polymicrobial infections [48–52]. These approaches enable rapid pathogen detection, with high specificity and sensitivity, and support multiplex assays for the simultaneous diagnosis of multiple pathogens. Yet, these approaches are relatively expensive for routine clinical use, requiring specialized equipment and training. Also, they cannot distinguish between viable and non-viable microbes and provide limited antimicrobial susceptibility information.

**Figure 1:**
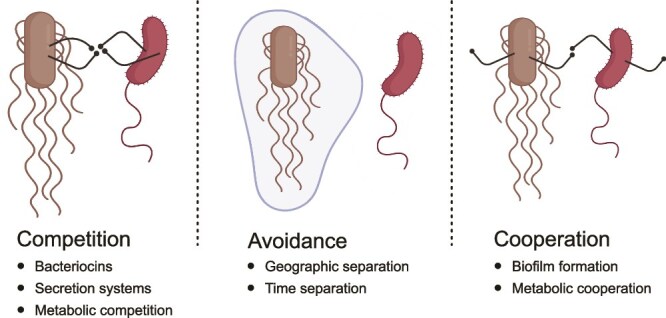
**Strategies for survival and growth in polymicrobial environments**. The figure illustrates the main strategies employed by bacteria in mixed communities: (1) bacterial competition, where organisms directly antagonize competitors through mechanisms such as production of bacteriocins, delivery of effectors by secretion systems, or metabolic competition for limited resources; (2) competition avoidance, where bacteria minimize direct competition through spatial or temporal separation; and (3) bacterial cooperation, where bacteria engage in mutually beneficial interactions including metabolite sharing, collaborative biofilm formation, and cross-feeding relationships. These strategies are not mutually exclusive, and bacteria often employ multiple approaches simultaneously depending on the environmental conditions and community composition.

Another useful approach is the use of immunological-based methods, which include antigen detection techniques, such as ELISA, and serological methods that target host antibody responses. Antigen detection techniques offer relatively rapid results and are suitable for automation; however, they may suffer from reduced sensitivity and specificity and cannot confirm organism viability, as they detect only microbial components [53, 54]. Serological methods are valuable for identifying infections by measuring host immune responses, but they are less effective for early diagnosis and often cannot differentiate between past and current infections [55]. Microscopy and mass-spectrometry methods both rely on prior microbe isolation but offer rapid results, which are based on phenotypic presentation [56, 57]. These methods have been optimized for the detection of a single pathogen and therefore require adaptation to multiplex platforms to effectively identify and characterize polymicrobial infections [58, 59].

In order to be valuable, diagnostic tools must be accurate (high specificity and sensitivity) and optimally deliver rapid results. They should also be cost-effective and accessible in multiple health care settings. For polymicrobial infections, diagnostic tools must enable simultaneous detection of all involved pathogens, regardless of their growth or metabolic requirements, while also determining their antimicrobial profile, allowing for proper treatment of these infections. While significant advancements in bacterial diagnostic methods have occurred in recent years, options for the diagnosis of polymicrobial infections remain limited, creating a critical gap between clinical need and available technology.

## Strategies to succeed in polymicrobial environments

Bacterial infections can stem from several contamination sources, including food and water, surfaces and objects, especially in healthcare settings, and environmental sources (soil, air) [60]. These sources may harbor single pathogens, but more commonly contain multiple pathogens that can be introduced simultaneously into the host. The presence of multiple bacteria in contaminated environments can drive diverse strategies that promote successful infections: (i) competition, (ii) competition avoidance, or (iii) cooperation (Fig. 1).

### Bacterial competition

Bacterial competition relies on the concept that only the most resilient and efficient strains will flourish in competitive environments. To achieve that, bacteria develop mechanisms to kill competitors or compete more effectively for nutrients or space [61]. This natural selection can enhance beneficial traits, like antibiotic resistance and metabolic efficiency. Studying bacterial competition led to the discovery of antimicrobials, as bacteria often produce compounds to inhibit competitors [62, 63]. Although pathogens with effective competitive strategies frequently dominate infections, leading to mono-infection, competitors may display defensive strategies, such as immunity proteins or altered targets to reduce susceptibility, allowing them to persist alongside the dominant pathogen. Furthermore, successful competition strategies by pathogens can affect the microbiome, disrupting colonization resistance and favoring polymicrobial infections.

#### Bacteriocins

Bacteriocins are small proteinaceous molecules that inhibit bacterial growth, allowing producers to outcompete other strains for resources and space [64]. Bacteriocins are highly diverse in structure, size, mode of killing, and mechanisms of production and secretion. Bacteriocins can be classified according to their target (membrane, DNA, RNA, proteins) or according to their function (pore-forming activity, nuclease activity, inhibition of peptidoglycan production, modulation of enzyme activity, quorum-sensing (QS) interference) [65].

Due to their ability to target specific bacteria and relatively high heat stability, bacteriocins have been investigated as food preservatives [66]. Recently, the potential of bacteriocins in targeting antibiotic-resistant pathogens has been explored. Commensal *E. coli* was shown to produce small bacteriocins, such as the microcin H47 toxin, that limit *Salmonella enterica* serovar Typhimurium and *E. coli* pathogenesis toward host cells [67, 68]. Nevertheless, most bacteriocins have narrow spectrum, typically inhibiting or killing genetically related bacteria, suggesting they are probably not major players in polymicrobial infections. However, recent studies reported that bacteriocins produced by Gram-positive bacteria exhibit broader spectrum, targeting other bacterial species or genera [69]. This suggests bacteriocins may play significant roles in bacterial interactions.

#### Bacterial secretion systems

**Figure 2:**
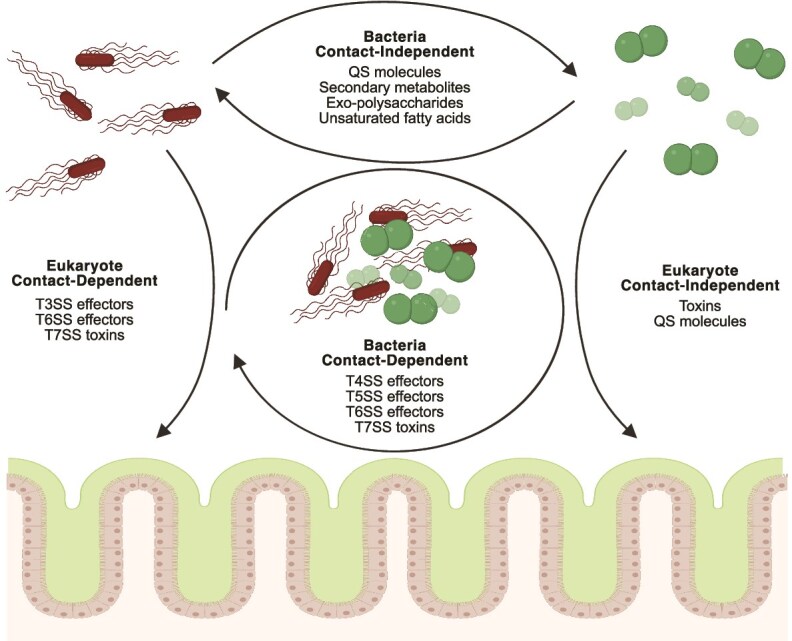
**Effectors used by bacteria to target other pathogens or host cells in polymicrobial infections**. Bacteria enhance their colonization and replication by manipulating host cells and competing against other microbes through the activity of various effectors. These effectors are delivered via **contact-independent** secretion, including the release of quorum sensing (QS) molecules, secondary metabolites, exopolysaccharides, and unsaturated fatty acids that influence neighboring bacteria, as well as secreted toxins and QS molecules that act toward eukaryotic cells. In contrast, **contact-dependent** secretion relies on direct contact and the use of secretion systems such as the T4SS, T5SS, T6SS, and T7SS to target other bacteria and T3SS, T6SS, and T7SS to deliver effectors into eukaryotic cells. These strategies contribute to bacterial competition, cooperation, and virulence in different environments.

Bacteria evolved secretion systems to deliver proteins, nucleic acids, and metabolites to the surrounding medium or directly into cells [70, 71]. Some of these secretion systems are essential for growth and survival in some environments, whereas others are dedicated to secreting components crucial for host infection or competition with nearby bacteria. The effectors of these secretion systems can act on various targets within the bacterial pathogens or the host cells they infect (Fig. 2). Among the various types of secretion systems (I-IX), four are particularly known for their roles in facilitating bacterial competition [71].

Type 4 secretion systems (T4SSs)—T4SSs are large protein complexes predominantly found in Gram-negative bacteria. T4SSs transport substrates directly from the cytoplasm of donor cells into the cytoplasm of recipients. T4SSs can interact with both eukaryotic and prokaryotic cells and can transfer DNA between bacteria or virulence proteins (effectors) into prokaryotic/eukaryotic cells. T4SSs facilitate bacterial competition by delivering toxic effectors into competitors within the same niche. These toxins employ diverse killing mechanisms, including enzymatic degradation of essential cellular components or membrane disruption. For instance, *Stenotrophomonas maltophilia*, a bacterium associated with medical device-related infections, induces contact-dependent killing of *E. coli*, *K. pneumoniae*, and *P. aeruginosa* by T4SS toxins [72]. A recent study of two soil bacteria, *Lysobacter enzymogenes* and *Pseudomonas fluorescens*, identified a mechanism through which T4SS promotes bacterial competition [73]. The translocated effector disrupts the production of QS molecules in *P. fluorescens*, leading to reduced biofilm formation and increased susceptibility to killing. Moreover, due to their well-known role in horizontal gene transfer, T4SS could potentially increase pathogen competition by horizontally transferring genes that could enhance or reduce bacterial virulence and survival during infection.

Type 5 secretion systems (T5SSs)—Protein secretion by T5SSs takes place in a two-step procedure and is autonomous—the secreted protein is responsible for its transport across the membrane. The first secretion step, across the inner membrane and into periplasmic space, is executed by the general bacterial secretion route (Sec pathway), an essential, ubiquitous, and universal export machinery. Sec transport is mediated by a short, cleavable amino-terminal signal peptide recognized by the Sec machinery [74]. The second step, through the outer membrane to the extracellular environment, is mediated by the beta-barrel domain at the C-terminus of the T5SS protein. This domain folds across the outer membrane to create a pore through which the secreted portion of the protein, the passenger domain, translocates to the bacterial surface. In most cases, the passenger domain is released after translocation, by cleavage between the passenger domain and the beta-barrel domain, which remains anchored in the outer membrane [75]. Passenger domains of T5SSs have a broad spectrum of functions, including adhesion, enzymatic activity, immune modulation, and even toxin production. T5SS substrates can act in their cell-surface adhered form (contact-dependent) or as secreted proteins (contact-independent). T5SSs are involved in bacterial competition by facilitating toxin delivery (nucleases, lipases, proteases) into neighboring bacteria, damaging them by degrading essential biomolecules. One of the most documented T5SS involved in competition is the Contact-Depended growth Inhibition (Cdi) system, CdiAB [76, 77]. CdiA has a C-terminal effector domain that is delivered into competitors by CdiB. Once inside cells, the effector degrades nucleic acids, inhibiting growth and, ultimately, causing death [78]. The specificity of these systems ensures that toxins selectively target competing species/strains, sparing those that express immunity proteins that neutralize effectors [79].

Type 6 secretion systems (T6SSs)—The T6SS is another contact-dependent killing mechanism employed by Gram-negative bacteria. T6SSs are needle-like structures that inject toxic effectors directly into target cells (prokaryotic or eukaryotic), making them versatile tools for bacterial competition and virulence [80–83]. Structurally, T6SSs resemble a phage tail or a contractile injection system with several key components (baseplate, sheath, needle). There are many examples of T6SS involvement in interbacterial interactions, including those that allow pathogens to outcompete commensals in their environment. However, only a few studies explored T6SSs in pathogen-pathogen interactions. A recent study revealed that *Aeromonas dhakensis*, a seafood bacterium, uses its T6SS to eliminate *Vibrio* competitors in shrimp [84]. Similarly, another study showed that T6SS effectors of *P. syringea* possess DNase activity, actively killing *E. coli* through DNA degradation [85]. Moreover, the T6SS of *Shigella sonnei* provides a competitive advantage against *S. flexneri* in mixed cultures [86]. By injecting toxins into competitors, T6SSs disrupt cell membranes, degrade cell walls, and interfere with essential processes, inhibiting growth or killing them [87]. This provides a competitive advantage in microbial communities and aids in establishing infections. Many pathogens, such as *V. cholerae* and *P. aeruginosa*, express T6SSs with high levels of antagonism against a broad range of commensals and pathogens, including *E. coli* and *S. enterica*.

Type 7 secretion systems (T7SSs)—The T7SS is found mostly in Gram-positive bacteria and has been implicated in nutrient acquisition, interbacterial competition, and virulence [88]. Given their limited similarity, T7SSs are classified into two subtypes; T7SSa in *Actinobacteria* and T7SSb in *Firmicutes*. While T7SSs are not as well-studied as other secretion systems, previous studies showed that some pathogens, such as *S. aureus* and *Enterococcus faecalis*, can promote the killing of competitors by delivering toxins through their T7SSs [89, 90].

### Metabolic competition

One of the most common ways microbes compete in polymicrobial environments is by fighting for nutrients. Because many bacteria share metabolic needs, competition for resources is intense. This leads to the evolution of metabolic traits that allow bacteria to bypass competition, often accessing less common nutrients that competitors cannot utilize for growth. Metabolic competition is widely recognized as a key mechanism by which the microbiome provides colonization resistance against invaders. Multiple pathogens also use this strategy when competing against the microbiome and other pathogens alike.

#### Metabolic competition in the gut environment

Ethanolamine is a gut nutrient derived from membrane phospholipids. Recently, the opportunistic pathogen *Klebsiella pneumoniae* was shown to utilize ethanolamine as carbon and nitrogen sources during gut colonization, an ability conferred by genes encoded within two *eut loci*, which are strongly induced during gut colonization [91]. Also, single and double mutants of *eut loci* showed defects in gut colonization in the presence of an intact gut microbiome. However, gut microbiome ablation by antibiotic treatment eliminated these differences, suggesting *eut loci* are involved in the ability of *K. pneumoniae* to outcompete the microbiome [91].

Besides *K. pneumoniae*, other pathogens use ethanolamine as a substrate during metabolic competition. An *eutC* mutant of *S. enterica* exhibited a significant competitive disadvantage in mouse co-infections with the wildtype strain [92]. *S. enterica* cannot efficiently utilize ethanolamine as a carbon source during fermentative growth *in vitro*. However, during anaerobic respiration in the presence of the alternative electron acceptor tetrathionate, *S. enterica* can grow on ethanolamine as the sole carbon source. The requirement for tetrathionate respiration also occurs *in vivo*, as an *eutC* mutant showed no competitive defect against the wildtype when *ttrA*, the gene encoding the tetrathionate reductase subunit A, was absent. Therefore, *S. enterica* uses anaerobic respiration in the gut to access a substrate that provides limited benefit to the predominantly fermentative microbial community of the anoxic mammalian gut [92]. Whether *K. pneumoniae* and *S. enterica* compete for ethanolamine *in vivo* remains unknown. However, that both pathogens devised mechanisms to access this nutrient suggests such competition likely occurs. Also, although considered a pathogen, *K. pneumoniae* can asymptomatically colonize the human gut. Under these conditions, *K. pneumoniae* may prevent *S. enterica* colonization by utilizing ethanolamine.

#### Metabolic competition in the CF lungs

CF is caused by mutations that impair chloride transport across the cell membrane, disturbing osmotic balance at the epithelial surface [93]. This results in dehydrated, thick mucus on the airway surfaces, favoring the attachment and proliferation of bacteria, which resist clearance. Polybacterial infections in CF commonly involve *S. aureus* and *P. aeruginosa*, highly proficient biofilm formers. A recent study demonstrated that *P. aeruginosa* secretes polysaccharides that specifically inhibit *S. aureus* growth, a finding confirmed using CF clinical isolates [94]. Recently, metabolic competition between these pathogens was studied using a synthetic CF medium [95]. Metabolomics was used to determine the nutrients consumed and metabolites produced by these pathogens. As expected, a vast overlap of metabolites consumed and produced was observed when different strains of the same species were compared. However, a sizable overlap was also observed in the metabolites consumed by both pathogens. Specifically, 15 metabolites, including beta-leucine, tryptophan, homoserine, indole-3-acrylic acid, serine, fructose, lactate, and glutamate, were degraded by all strains tested, indicating they can be reliable sources of carbon and energy for both species. Although these experiments were performed in monocultures, the vast metabolic overlap identified likely has implications during co-infection *in vivo*.

Aside from the competition between *S. aureus* and *P. aeruginosa* for carbon and energy sources, *P. aeruginosa* can also directly alter *S. aureus* metabolism, with implications for competition. A co-culture model of *S. aureus* and *P. aeruginosa* on CFTR-deficient human bronchial epithelial cells showed that *P. aeruginosa* shifts *S. aureus* metabolism from aerobic respiration to fermentation [96]. mRNA sequencing revealed *P. aeruginosa* strongly upregulated *S. aureus* fermentation genes (≥10-fold), an effect reproduced with *P. aeruginosa* cell-free supernatants. In contrast, the effect of *S. aureus* on *P. aeruginosa* gene expression was minimal, with only one gene being differentially regulated. Production of 2-heptyl-4-hydroxyquinoline N-oxide (HQNO) and the siderophores pyoverdine and pyochelin by *P. aeruginosa* was responsible for this phenomenon. These mechanisms of competition between *S. aureus* and *P. aeruginosa* have biological relevance. During lung infections of CF patients, although both *S. aureus* and *P. aeruginosa* are present, a succession is commonly observed, where *S. aureus* dominates the CF microbial community earlier in life, with *P. aeruginosa* taking over later on.

### Competition avoidance

Competitive avoidance describes a state where bacterial species that use similar resources coexist by avoiding one another. This can be achieved by maintaining physical or temporal separation, reducing direct competition. In some of the examples below, competition avoidance may seem like an active process, whereas in others it may be a simple consequence of extraneous factors. Whether active or passive, this phenomenon allows for a balance where multiple species can thrive within the same environment without outcompeting each other to extinction.

#### Geographic separation

One strategy to avoid competition is to geographically divide the niche into smaller areas. Although this strategy was mainly demonstrated among fungal pathogens [97, 98], there are examples for geographic separation between bacterial pathogens and commensals. Bacterial populations can create physical separation by producing compounds, such as extracellular polysaccharides (EPS), polyamides, and extracellular DNA, that define distinct microenvironments forming physical barriers between cells [99]. One example is *Cutibacterium acnes*, a prevalent member of the skin microbiota that comprises ~92% of bacterial populations of oily skin regions. *C. acnes* was shown to maintain intraspecies diversity by promoting spatial segregation of different strains across distinct skin pores [100]. Each pore is colonized by a single strain, although multiple strains are present in neighboring pores. No genetic adaptations were found to drive this phenomenon; pore anatomy and physiology seem to induce bottlenecking, avoiding direct competition between different *C. acnes* strains, and enhancing overall strain diversity. Although *C. acnes* is generally a commensal, it can cause significant prosthetic joint infections in humans, and it is possible that geographic separation also occurs during polymicrobial infections [101].

Spatial separation between *P. aeruginosa* and *S. aureus* through a cell-free physical barrier has also been described, a phenomenon derived from the production of phenol-soluble modulins (PSMs) by *S. aureus* colonies [102]. PSMs form amyloid fibrils around the colonies, deflecting the surfactant flow created by *P. aeruginosa* and steering its swarms away. Recently, it was also shown that *S. aureus* produces extracellular surfactants that affect *P. aeruginosa* motility, enabling it to spread across semi-solid surfaces that would otherwise restrict its movement [103]. Using another species’ surfactants would, in theory, allow *P. aeruginosa* to access different environmental niches within the host while avoiding competition. Another study reported that *V. cholerae* can produce an EPS that functions as an armor that protects it from T6SS-mediated attacks by heterologous bacterial species, enabling coexistence in the same niche [104].

Physical separation between pathogens can also be achieved through their natural preference for intracellular *versus* extracellular environments. A recent study that examined the prevalence of diarrhoeal pathogens in pediatric samples found high numbers of bacterial poly-infections of intracellular-extracellular pathogens, such as the triplets *Shigella*, Enteroinvasive *E. coli* (EIEC), and EPEC; Enteroaggregative *E. coli* (EAEC), EPEC and *Campylobacter*; and the pair EAEC and *Salmonella* [105]. Although we are unaware of evidence that the presence of a competitor in the extracellular environment could drive invasion by an intracellular pathogen, the ability of intracellular pathogens to physically separate themselves from extracellular competitors may allow them to survive and multiply in what would be an otherwise hostile environment due to pathogen competition.

#### Time separation

Sequential bacterial infections are co-infections that occur at slightly different times. Until recently, these infections were considered to stem from the arrival of distinct infectious agents at different times. The first (primary) infection was considered to facilitate sequential infections (secondary or super-infections) by harming the immune response, altering the native microbiome, or disrupting host tissues. However, recent models suggest these infections might also stem from the simultaneous arrival of multiple pathogens into the host, but with clinical manifestations occurring at different times, as a means to support both infections. In most sequential infections, the primary pathogen is a virus [106, 107]. However, specific pairs of bacterial pathogens were reported to occur in sequential infections. For example, following *M. tuberculosis* infections, superinfection with *H. influenzae* or *S. aureus* is common [108]. A high incidence of *P. aeruginosa* infection (23.5%) was detected among patients receiving the antibiotic tigecycline, mainly used to treat nosocomial infections by MRSA, penicillin-resistant *S. pneumoniae*, vancomycin-resistant *Enterococcus* spp., and *Enterobacteriaceae*, and *P. aeruginosa* likely appears as a late-stage co-infecting pathogen in these infections [109].

Time separation between pathogens was recently reported between *V. cholerae* and EPEC. *V. cholerae*, the causative agent of cholera, causes an estimated 3-5 million cases annually, with ~100,000 deaths. In endemic countries, about half of the deaths occur in young children [7, 110]. EPEC is a prevalent diarrheal pathogen that leads to pediatric and persistent diarrhea [111]. While EPEC-related illness has not been highly prevalent in developed countries since the 1950’s, it is recently reemerging, with severe disease outcomes in the community and hospital settings [111]. *V*. *cholerae* and EPEC share the same route of infection, source of infection (contaminated water or food), and symptoms (gastroenteritis). Additionally, both display tropism toward the small intestine and are often found together in clinical samples. A recent study showed EPEC senses the presence of *V. cholerae* and adjusts its virulence accordingly to coordinate successful host colonization [112]. By measuring the concentration of the main *V. cholerae* QS molecule (Cholerae Autoinducer-1 (CAI-1)), EPEC monitors the population size and virulence status of *V. cholerae*. To optimize its infection for times of minimal competition, EPEC upregulates its virulence when it detects high CAI-1 concentrations, indicating downregulation of *V. cholerae* virulence and their preparation to leave the host [112]. These results were unexpected, as CAI-1 was previously considered an exclusively intra-genus (*Vibrio*) signal [113, 114]. A follow-up study demonstrated that production of indole by members of the gut microbiome, like *Bacteroides thetaiotaomicron*, disrupt EPEC’s ability to sense CAI-1 and time its infection to periods of lower competition with *V. cholerae* [115]. These findings suggest that bacterial interactions involving members of the gut microbiome may also affect the time separation displayed by *V. cholerae* and EPEC during polymicrobial infection.

Co-infections of the sexually transmitted bacteria *Neisseria gonorrhoeae* and *Chlamydia trachomatis*, which infect the epithelium of the endocervix in women and the urethra in men, also exemplify time separation. Researchers found that, during infection, when *C. trachomatis* is exposed to extracellular *N. gonorrhoeae* at mid-cycle, it continues to grow but does not transition into the infectious elementary body state as fast as it does during mono-infections [116]. This slower kinetics of bacterial development results in delayed release of progeny, which can benefit *C. trachomatis* by timing the release of infectious elementary bodies to periods when the environment is less hostile.

### Pathogen cooperation

Microbial cooperation enhances the fitness of all microbes by enabling them to share resources, create protective structures that promote survival under environmental stresses, like antibiotics, and perform complementary metabolic functions. Microbial synergy can occur between pathogens during infection, allowing them to colonize more effectively. For instance, one pathogen might create conditions that favor the growth of another, such as altering the local environment to reduce immune responses or providing essential nutrients. These cooperative interactions can contribute to the persistence and virulence of pathogens, leading to harder-to-treat infections [117, 118].

#### Biofilm formation

Biofilms have increased resistance to antimicrobial agents and immune responses [119, 120]. Generally, monobacterial biofilms are more commonly associated with acute infections, such as UTIs or uncomplicated skin infections. In contrast, biofilms containing multiple species are more frequently observed in chronic and harder-to-treat infections. Polymicrobial biofilms are frequently found in periodontitis, otitis media, diabetic foot wounds, burn wounds, and infections of CF patients. These polymicrobial biofilms provide an optimal environment for different species to interact antagonistically, additively, or synergistically, impacting their persistence and stability during infection [31, 121]. Consistent with their detrimental effect on treatment, polymicrobial infections in chronic wounds delay closure and promote antibiotic resistance compared to mono-infections [122, 123]. Furthermore, polymicrobial biofilms were shown to initiate and aggravate CF and burn infections [124, 125]. By establishing multi-species biofilms, bacteria gain competitive advantages, including antimicrobial resistance, metabolic cooperation, QS regulation, and higher chances of horizontal gene transfer.

Two common partners in polymicrobial infections, *P. aeruginosa* and *S. aureus*, have been shown to cooperate in an *in vitro* wound model by displaying enhanced antibiotic tolerance [126]. Enhanced resistance is dependent on components of the host matrix and extracellular polymeric substances produced by both species. The increased aggregation and tobramycin resistance exhibited by *P. aeruginosa* is dependent on the interaction between *S. aureus* protein A and *P. aeruginosa* Psl (an exopolysaccharide) [127]. In contrast, *S. aureus* senses HQNO produced by *P. aeruginosa*, forming small colony variants, which are aminoglycoside-resistant and associated with persistent infections [127, 128]. It has also been shown that *S. aureus* displays passive resistance to ampicillin by becoming closely surrounded by a dense layer of ampicillin-resistant *P. aeruginosa* [129].

The spatial organization of biofilms can modulate virulence and overall progression of infection. Cooperative interactions in biofilms have been classified as derived from physical contact or chemical signaling [130]. One example of contact-dependent interaction that segregates species in biofilms happens between *P. aeruginosa* and *S. aureus*. In CF, several variants of *P. aeruginosa* overproduce the polysaccharide alginate, enhancing biofilm formation with *S. aureus* and *B. cepacia* and leading to highly structured biofilms with increased antibiotic resistance [131, 132]. This species organization impacts biofilm stability and persistence, consequently affecting the success of the infection. In another case of contact-dependent interaction, two periodontal pathogens, *Treponema denticola* and *Porphyromonas gingivalis*, have been shown to form thicker biofilms when co-cultured [133], a phenomenon dependent on the expression of membrane-associated proteins or adhesins, gingipains, by *P. gingivalis.*

Within polymicrobial biofilms, different species can detect one another by sensing metabolites. These metabolites include byproducts produced during cross-feeding or dedicated signals, such as QS molecules. Metabolite-based detection has been shown to occur between two opportunistic pathogens commonly associated with otitis media, *Moraxella catarrhalis* and *H. influenzae* [10]. Although *M*. *catarrhalis* cannot produce QS autoinducer-2 (AI-2), it responds to it. Co-culturing *M*. *catarrhalis* with *H. influenzae*, which produces AI-2, results in more robust biofilms, which are more tolerant to antimicrobials. In an animal model of otitis media, the interaction between these species was shown to support higher levels of *M*. *catarrhalis* growth in an AI-2-dependent fashion*.* A similar pattern was observed for *P. aeruginosa*; although *P. aeruginosa* cannot produce AI-2, it enhances virulence factor expression in CF lung infections in response to AI-2 from co-infecting bacteria [134].

#### Metabolic cooperation

Metabolic cooperation can promote polymicrobial interactions, as seen in *E. faecalis* and *E. coli*, which are commonly found in urinary catheters and wound infections. Co-culturing *E. faecalis* and *E. coli* enhanced their ability to form biofilms under iron-limiting conditions [135]. The underlying mechanism involves L-ornithine production by *E. faecalis*, triggering *E. coli* to produce the siderophore enterobactin, boosting iron acquisition, bacterial growth, and biofilm formation. In a mouse wound infection model, *E. coli* reached significantly higher colonization levels during co-infections.

Synergistic interactions based on metabolic cross-feeding also occur during periodontitis. *Aggregatibacter actinomycetemcomitans* is an oral pathogen found in patients with aggressive periodontitis. These infections are often polymicrobial, with *Streptocoocus gordonii* frequently present. It has been shown that an *A. actinomycetemcomitans* mutant that cannot metabolize lactose can cause infections in monoculture, but cannot synergize with *S. gordonii* in polymicrobial infections. Because *A. actinomycetemcomitans* utilizes lactate as a carbon source and *S. gordonii* produces large amounts of lactate during carbohydrate metabolism, *A. actinomycetemcomitans* cross-feeds on lactate to enhance the severity of the disease [136]. In turn, *A. actinomycetemcomitans* neutralizes *S. gordonii*-produced hydrogen peroxide via catalase. Hydrogen peroxide also upregulates *A*. *actinomycetemcomitans apiA* expression; *apiA* encodes a protein that binds human factor H, a regulator of the alternative complement pathway [137]*.* This interaction increases resistance to immune-mediated killing by *A. actinomycetemcomitans* when co-cultured with *S. gordonii*.

## Targeting pathogen interactions for therapy

**Figure 3:**
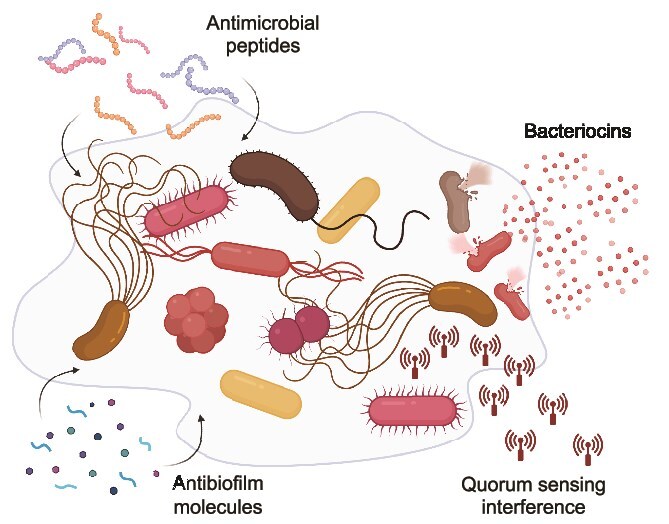
**Therapeutic strategies targeting polymicrobial infections**. The illustration depicts a polymicrobial biofilm and various therapeutic approaches that can be used alone or in combination with conventional antibiotics to combat these complex bacterial communities. Antibiofilm molecules may target the extracellular matrix, including matrix-degrading enzymes and dispersal agents that compromise biofilm structural integrity. They may also interfere with biofilm maturation processes. Bacterial cells within the biofilm can be targeted by antimicrobial peptides, which disrupt bacterial cell membranes, and bacteriocins, which provide diverse antimicrobial activity. Quorum-sensing inhibitors interfere with bacterial cell-cell communication through multiple mechanisms, including degradation of signaling molecules and receptor antagonism. This multi-modal approach addresses the various aspects of the polymicrobial pathogen community, potentially offering more effective treatment strategies for polymicrobial infections compared to conventional antibiotics alone.

Current treatments for infections typically target individual pathogens despite the high prevalence of polymicrobial infections. This narrow focus overlooks the complex dynamics often present in mixed infections. Treatment of polymicrobial infections can be more challenging because different bacterial species often display different antimicrobial susceptibility patterns. Furthermore, synergistic interactions enhance biofilm formation, adding an extra layer of resistance to many treatments [128, 138, 139]. However, the presence of multiple pathogens does not always result in an additive virulence effect. This was demonstrated using a *Galleria mellonella* model, where the primary determinant of virulence in a polymicrobial infection was found to be the most virulent species [140]. In certain pathogen combinations, the presence of a co-infecting pathogen actually reduced the fitness of the primary pathogen. This was specific to certain bacterial pairs, highlighting the complexity of predicting the outcome of polymicrobial infections. Nevertheless, developing effective treatment strategies for polybacterial infections remains crucial and requires a multi-faceted approach (Fig. 3). Two main antimicrobial strategies can be employed: broad-spectrum antimicrobials targeting multiple bacterial species simultaneously or combination therapies integrating several narrow-spectrum drugs, each selectively targeting one pathogen. Because many polybacterial infections involve biofilms, which are significantly more resistant, anti-biofilm drugs can be deployed to target these resilient infections. Furthermore, targeting bacterial communication through QS inhibitors represents an innovative approach that could prevent bacterial coordination and reduce virulence. Anti-biofilm and anti-QS therapeutics could be deployed either independently or in combination with antibiotics. Disrupting these virulence mechanisms may boost the host's immune response to eradicate the pathogens on its own or make bacteria more susceptible to antibiotics. While these strategies offer promising alternatives to conventional treatments, their clinical potential remains largely underexplored and warrants further investigation.

### Bacteriocins

Bacteriocins emerged as potent antimicrobials for the treatment of mono-infections. Traditionally, bacteriocins are considered to have a narrow spectrum of activity, primarily targeting genetically related bacteria. As a result, their use is largely confined to the food industry. However, recent studies demonstrated that several bacteriocins are effective against a wide range of pathogens [141–143]. For example, lactobacilli produce bacteriocins that interfere with cell wall biosynthesis and induce the formation of pores in *S. aureus* membranes [144]. To counter this activity, *S. aureus* produces bacteriocins (staphylococcin Au-26, Bac1829, BacR1, aureocin A53) that inhibit lactobacilli growth [144]. The combination of multiple bacteriocins or the use of bacteriocins with antibiotics demonstrates the highest antibacterial efficacy [145, 146]. Bacteriocins are also used during competition between *S. aureus* and *Corynebacterium* spp., microbes associated with skin, nasopharynx, and catheter-related infections. Several bacteriocins with activity against *Corynebacterium* spp. (Bac 1829, aureocin A70, aureocin A53, and staphylococcin 188) are secreted by *S. aureus* [147–149]. However, despite their potential as antimicrobial agents, nisin is the only bacteriocin approved for use by regulatory agencies, and even then, its use is limited to food preservation. It would be valuable to investigate the impact of bacteriocins on polymicrobial infections, as they may influence bacterial dynamics during infection and improve pathogen eradication.

### Antibiofilm molecules

Because biofilm formation offers considerable advantage to polymicrobial infections, disrupting biofilms will likely profoundly impact infections. There are two strategies for biofilm disruption: preventing their formation or dismantling them after they have formed. Biofilm prevention is considered easier and can be done by changing materials used in medical devices, coating them with anti-adherence compounds, or interfering with signaling pathways that induce biofilms [150]. The most common strategy is creating materials with reduced adhesion properties, including materials with enhanced smoothness, reduced hydrophobicity, and low surface energy. These properties can be achieved by selecting appropriate materials or applying coatings imparting the necessary characteristics. For example, the extremely hydrophilic 2-methacryloyloxyethyl phosphorylcholine polymers can significantly decrease bacterial pathogen attachment and prevent biofilm formation [151]. Moreover, coating with silicone rubber or surfactants reduces interfacial tension and the ability of bacteria, like *S. aureus*, to form biofilms [152, 153]. Another strategy to disrupt biofilms involves targeting signaling pathways essential for their development. These include QS molecules and nucleotide second messengers that reduce bacterial motility, promote surface attachment (such as pilus formation), or enhance biofilm matrix production (EPS, cellulose, curli fibers). The regulation of biofilm formation via nucleotide second messengers mainly includes (p)ppGpp, c-di-AMP, and c-di-GTP [154]. Interference with these secondary metabolites can be achieved by reducing their synthesis or enhancing their degradation. For example, small molecules that antagonize the activity of diguanylate cyclases or diadenylyl cyclases, which synthesize c-di-GMP and c-di-AMP, respectively, inhibit biofilm formation [155, 156].

Dismantling mature biofilms is considered more challenging but can be accomplished by physical or biochemical methods [157]. Physical methods, such as ultrasound and magnetic field application, are effective for eradicating biofilms on medical devices and patient-contact surfaces but unsuitable for treating infections directly. Biochemical methods, including phage lysins, degradative enzymes, and microbial metabolites, are more appropriate for treating patients. Several enzymes effectively metabolize components of the biofilm and its matrix. These include EPS hydrolytic enzymes, proteases that cleave biofilm-associated proteins, and DNases that degrade extracellular DNA (eDNAs) [158]. A combination of multiple antibiofilm agents showed enhanced effectiveness against biofilms of mixed pathogenic species (*L. monocytogenes* with *E. coli* or *P. fluorescens*) [159]. Despite significant potential, only a few anti-biofilm agents have been evaluated in clinical trials, and their full therapeutic potential has yet to be explored [160].

Although individual strategies show promise in disturbing biofilms, combining antibiofilm and antibacterial drugs may be more effective by targeting both biofilm structure and the cells within. Often, results demonstrate enhanced activity at lower doses, reducing the risk of resistance and side effects [161]. Acting on biofilm EPS can effectively reduce tolerance and the damage caused by the immune responses. A recent study showed that a combination of trypsin and DNAse I, both of which degrade EPS, effectively eradicates dual-species biofilms of *P. aeruginosa* and *S. aureus* [162]. In another study, researchers screened a small molecule library for antagonists of PQS, a quinolone-dependent QS system that controls virulence and biofilm production in *P. aeruginosa.* One antagonist, quinazolinone (QZN), successfully eradicated *P. aeruginosa* and *S. aureus* mixed biofilms in combination with tobramycin. These findings are particularly interesting given that *P. aeruginosa* is known to protect *S. aureus* from tobramycin-induced killing by promoting the formation of small colony variants [163]. Recently, a combination of phages and gentamicin was shown to effectively kill *P. aeruginosa* and *S. aureus* mixed biofilms using a wound model on artificial dermis [164]. Simultaneous and multiple consecutive doses were required to avoid the emergence of resistance to this treatment.

### Antimicrobial peptides

Antimicrobial peptides (AMPs) have been described as potential alternatives to treat biofilm-associated polymicrobial infections (reviewed in [139]). AMPs have several advantages compared to conventional antibiotics, displaying broad-spectrum antimicrobial and antibiofilm activity with a shorter incubation time and at similar or lower concentrations than antibiotics. Furthermore, these molecules often exhibit anti-inflammatory properties, speeding healing. In one study, a short synthetic AMP, DRGN-1, inhibited monomicrobial and polymicrobial biofilms of *P. aeruginosa* and *S. aureus* [165]. Using a mouse wound model, the peptide was shown to reduce the colonization of both species and also stimulate keratinocyte migration, accelerating wound closure. More recently, a short synthetic peptide, K6, showed bactericidal activity against *P. aeruginosa* and *S. aureus* polymicrobial biofilms in a mouse model of persistent infection [166]. In addition, K6 exhibited higher antibiofilm activity than gentamicin, inducing bacterial membrane permeability by forming nanostructured micelles. A study using a mouse model of pneumonia with multi-drug resistant *P. aeruginosa* and *A. baumannii* reported that pre-treatment of mice intravenously with the AMP tachyplesin III effectively prolonged survival and reduced bacterial counts in bronchoalveolar lavage fluid, even compared to meropenem-treated mice [167]. An additional synthetic AMP, Nal-P-113, showed bactericidal activity against polymicrobial biofilms of *S. gordonii*, *F. nucleatum*, and *P. gingivalis* at lower concentrations than penicillin or metronidazole [168]. Although promising, the use of AMPs has limitations, including potential for toxicity, susceptibility to protease degradation, low stability at physiological salt concentrations, sequestration by components of body fluids, and high costs.

### QS interference

QS is a key bacterial communication strategy that regulates population behaviors, host-microbe interactions, and virulence, prompting the evolution of mechanisms of QS interference as mechanisms of competition [169]. Indeed, the ability of microbes to interfere with QS of competitors, termed quorum quenching, is extensively described [170]. This can occur, for instance, through the degradation of QS signals, typically performed by acylases and lactonases. *P. aeruginosa*, including human clinical isolates, can grow in 3-oxododecanoyl-homoserine lactone (3OC12HSL), the primary QS signal of this species, as the sole source of carbon and energy [171]. The acylase enzyme involved in this activity, PvdQ, is also required to mature pyoverdine, an important virulence factor of *P. aeruginosa*. Indeed, inhibition of PvdQ is protective against *P. aeruginosa* infection in a *G. mellonella* model [172]. Therefore, QS enzymes have essential roles in *P. aeruginosa* virulence. Although, to our knowledge, the role of this enzyme in microbial competition has not been directly demonstrated, its ability to interfere with QS could have consequences during co-infections, such as those occurring in the CF lungs. In this complex microbial community, other AHL-producing pathogens, such as *Burkholderia cenocepacia*, are commonly found and use QS in their pathogenesis [173]. Two acylases of *P. aeruginosa*, PvdQ and QuiP, allow the degradation of multiple types of AHLs [171, 174]. In addition, AhlD, a lactonase from *Arthrobacter* sp. with orthologs in several bacteria, including the human pathogen *K. pneumoniae*, was previously described [175]. Also, lab-engineered variants of the SsoPox lactonase showed anti-virulence activity against multiple human pathogens, including *A. baumannii, P. aeruginosa*, and *B. cepacia*. Biofilm formation by these pathogens and toxicity towards macrophages by *P. aeruginosa* were significantly dampened by lactonase treatment [176]. Besides acylases and lactonases, a third class of enzymes, the oxidoreductases, interfere with AHL signaling. Although oxidoreductases do not effectively degrade AHLs, they chemically modify them by oxidizing or reducing acyl chains. This can alter signal specificity and, therefore, activity. Although this has been investigated mostly in environmental and plant-associated microorganisms, these enzymes were found in *Burkholderia*, a genus encompassing several human pathogens [177].

## Conclusions

Historical focus on single-pathogen infections has underestimated the clinical relevance of polymicrobial infections until recently. It is now evident that bacterial pathogens frequently exist within complex communities, both within the host and in environmental reservoirs. This has driven the evolution of sophisticated interactions, encompassing sensing, communication, competition, and evasion strategies, often contributing to increased infection persistence and treatment resistance. Understanding the intricate balance between antagonistic and synergistic interactions among pathogens can revolutionize therapeutic and preventative strategies. Investigations of pathogen interactions within the context of polymicrobial infections should be prioritized to fully realize this potential. This includes improving microbial isolation procedures for different clinical specimens and applying diagnostic tools to identify co-infections correctly. Once pathogens involved in polymicrobial infections are identified, the mechanisms by which these interactions facilitate pathogen establishment, persistence, and disease severity can be elucidated. Specifically, studies should focus on defining the molecular basis of interspecies communication, as understanding how pathogens signal and respond to each other is crucial to identifying potential targets for intervention. Additionally, characterizing the dynamics of competitive and cooperative interactions will assist in determining the factors that influence the outcome of these interactions, providing insights into the stability and resilience of polymicrobial communities. Studies should also focus on the role of the host environment, as understanding how host factors influence pathogen interactions and contribute to disease pathogenesis is essential to develop therapies. Finally, future studies that explore the impact of polymicrobial interactions on antimicrobial resistance are needed. Assessing the contribution of interspecies interactions to the evolution and dissemination of resistance is critical for combating the growing threat of antibiotic resistance. The development and validation of *in vitro* and *in vivo* models are essential to accurately replicate the complex relationships observed in polymicrobial infections. These models should incorporate relevant environmental and host factors to ensure translational relevance. Ultimately, the accumulated knowledge of pathogen interaction strategies will enable the development of targeted interventions. Strategies such as disrupting cooperative mechanisms or exploiting competitive interactions could provide novel avenues for combating polymicrobial infections. This paradigm shift, from targeting single pathogens to modulating interspecies interactions, holds immense promise for improving patient outcomes and addressing the challenges posed by complex infectious diseases.

## Data Availability

Data sharing is not applicable to this article as no datasets were generated or analyzed during the current study.
